# Small-sized colorectal cancer cells harbor metastatic tumor-initiating cells

**DOI:** 10.18632/oncotarget.22392

**Published:** 2017-11-11

**Authors:** Lei Mu, Kaiyu Huang, Yibing Hu, Chang Yan, Xiaolan Li, Deding Tao, Jianping Gong, Jichao Qin

**Affiliations:** ^1^ Department of Surgery, Tongji Hospital, Tongji Medical College, Huazhong University of Science and Technology, Wuhan 430030, China; ^2^ Molecular Medicine Center, Tongji Hospital, Tongji Medical College, Huazhong University of Science and Technology, Wuhan 430030, China

**Keywords:** colorectal cancer, tumor-initiating cells, metastasis, cell size, YAP1

## Abstract

Colorectal cancer (CRC) is heterogeneous and contains different-sized cells. Recent studies have shown that tumor-initiating cells (TICs) are involved in cancer initiation, recurrence and metastasis. However, connections between cancer cell size and stem-like properties are largely unknown. Here we purified large- and small-sized CRC cells by fluorescence-activated cell sorting (FACS) based on forward scatter (FSC), and demonstrated that small CRC cells possess higher holoclone- and sphere-forming capacity *in vitro*, tumor-initiating capacity *in vivo* and form more lung metastases compared with large CRC cells. Furthermore, we found that down-regulated YAP1 (yes-associated protein 1) decreased tumor-initiating and metastatic capacity in small CRC cells but not in large CRC cells. More importantly, our results showed that the expression of YAP1 positively correlated with the poor prognosis in CRCs. Collectively, our findings suggest that small CRC cells enrich for metastatic TICs, and YAP1 is one of the potential therapeutic targets of metastatic TICs, the small CRC cells.

## INTRODUCTION

Colorectal cancer (CRC) is the third most common cause of death from cancer, and CRC patients typically die due to tumor progression and metastatic lesions [1]. CRC is heterogeneous, manifesting variegated cellular morphologies and histopathological presentations. Experimental evidence for the existence of tumor-initiating cells (TICs) with metastatic capacity or metastatic TICs was recently shown in human CRCs [2–5].

CRCs contain different-sized cancer cells, but the regulation of cell size remains poorly understood. The cells possess different sizes when locate in different cell cycle phase during cell cycle progression [6]. In one same cell cycle, the cells in G_0_/G_1_ phase are generally smaller than the cells in S phase, and are much smaller than the ones in G_2_/M phase [7, 8]. However, in different cell cycles, the cells even in same cell cycle phases (i.e., G_0_/G_1_ phase) have varied cell sizes [9, 10]. In addition, numerous studies have clearly shown that mammalian adult stem cells are generally smaller than differentiated cells [11–15], however, whether small-sized cancer cells enrich for TICs (i.e., cancer stem cells) is not completely clear, particularly in CRCs.

Metastasis involves a multi-step process known as the invasion-metastasis cascade, which involves the outgrowth of the local primary tumors, intravasation of these tumor cells into the circulatory system and extravasation through vascular walls into the parenchyma of distant tissues [16, 17]. In the above events, the local primary cancer cells need to undergo both intravasation and extravasation and thereby enter into the parenchyma of distant tissues, and small-sized cancer cells are considered to easily pass through either blood or lymphatic vessels due to their cell size. However, whether small-sized cancer cells are prone to metastasize is also unknown. Herein, we separate CRC cells into the subpopulations of large- and small-sized cells, and investigate whether small-sized CRC cells possess cancer stem-like properties and metastatic capacity.

## RESULTS

### Different-sized CRC cells can be prospectively sorted out using FACS

**Figure 1:**
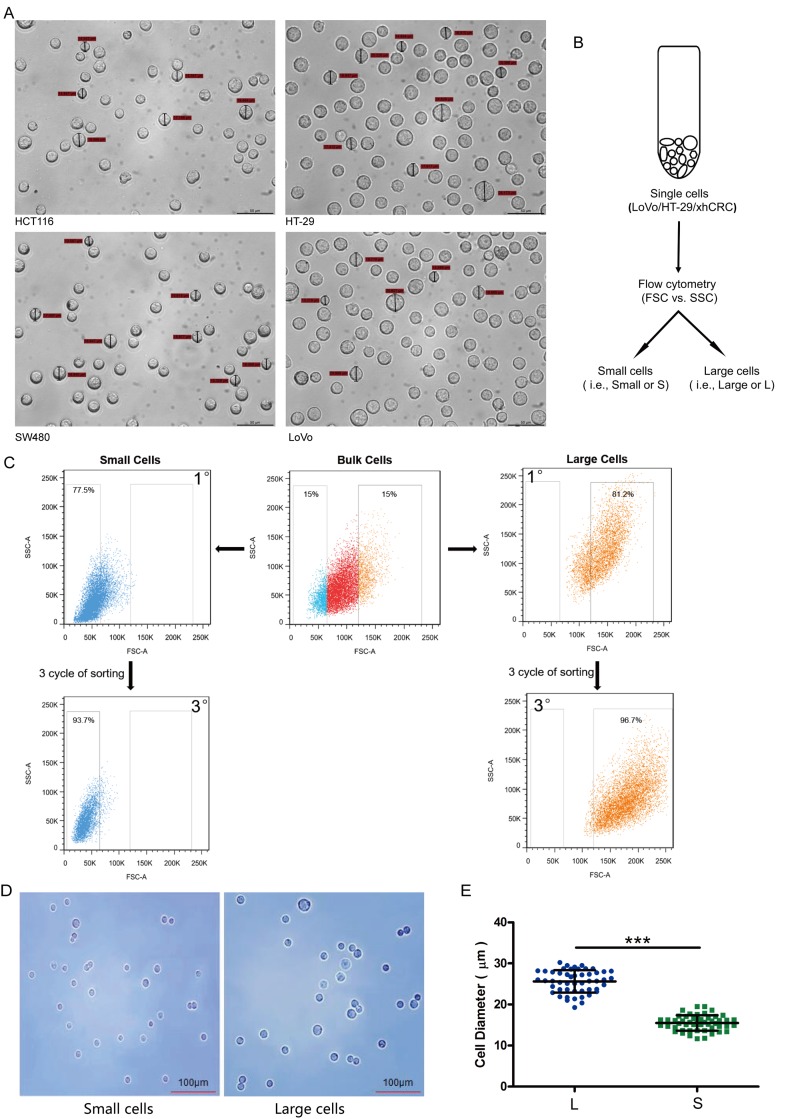
Large and small CRC cells can be prospectively sorted out by fluorescence-activated cell sorting (FACS) **(A)** Cell size of the cultured cells in CRC cell lines (i.e., HCT116, SW480, LoVo and HT-29) was measured under microscope. **(B)** Schematic of large and small CRC cells sorting. **(C)** Post-sorting analysis of the sorted large and small CRC cells. **(D)** Sorted cells were plated on glass cover slides and cell morphology was observed under microscope. **(E)** Diameters of sorted large cells (i.e., L) and small cells (i.e., S) were measured under microscope. Data are presented from triple experiments; mean ± SD, ^*^P< 0.05, ^**^P< 0.01, ^***^P< 0.001.

In order to determine whether CRC cells are able to sort out different-sized cells, we first measured the size of the cultured cells in widely used CRC cell lines, e.g. HCT116, SW480, LoVo and HT-29. As demonstrated in Figure 1A, the intratumor heterogeneity of cell size in LoVo and HT29 cells is high, and the two cell lines were thus used to sort out the large and small cells in subsequent experiments. Furthermore, recent studies have shown that patient-derived xenografts may retain the heterogeneity of their originating tumors, we also used one established patient-derived CRC xenografts (xhCRC) in our lab, which derived from a female patient with Duke C colorectal adenocarcinoma [18–20].

To separate large and small CRC cells (i.e., LoVo and HT29), we used fluorescence-activated cell sorting (FACS) to purify out the top 15% (i.e., large cells) and bottom 15% CRC cells (i.e., small cells) based on the value of Forward scatter (FSC), which measures the size of the cells (Figure 1B and 1C). To purify out large and small xhCRC cells, we first processed the xenograft tumors into single cell suspensions, and then sorted out two cell subpopulations (i.e., EpCAM^+^FSC^high^ and EpCAM^+^FSC^low^). The purity of large and small cells was ~97% and ~94% upon 3 cycles of sorting, respectively (Figure 1C). In order to further confirm that the purity of large and small cells, we observed the morphology and measured the diameters of the sorted cells under microscope. As shown in Figure 1D and 1E, the diameters of sorted large cells were significantly different from the ones of sorted small CRC cells (*P*<0.001). These results clearly indicate that purified large and small CRC cells are capable of being prospectively sorted out using FACS.

### Purified small CRC cells enrich for TICs

**Figure 2:**
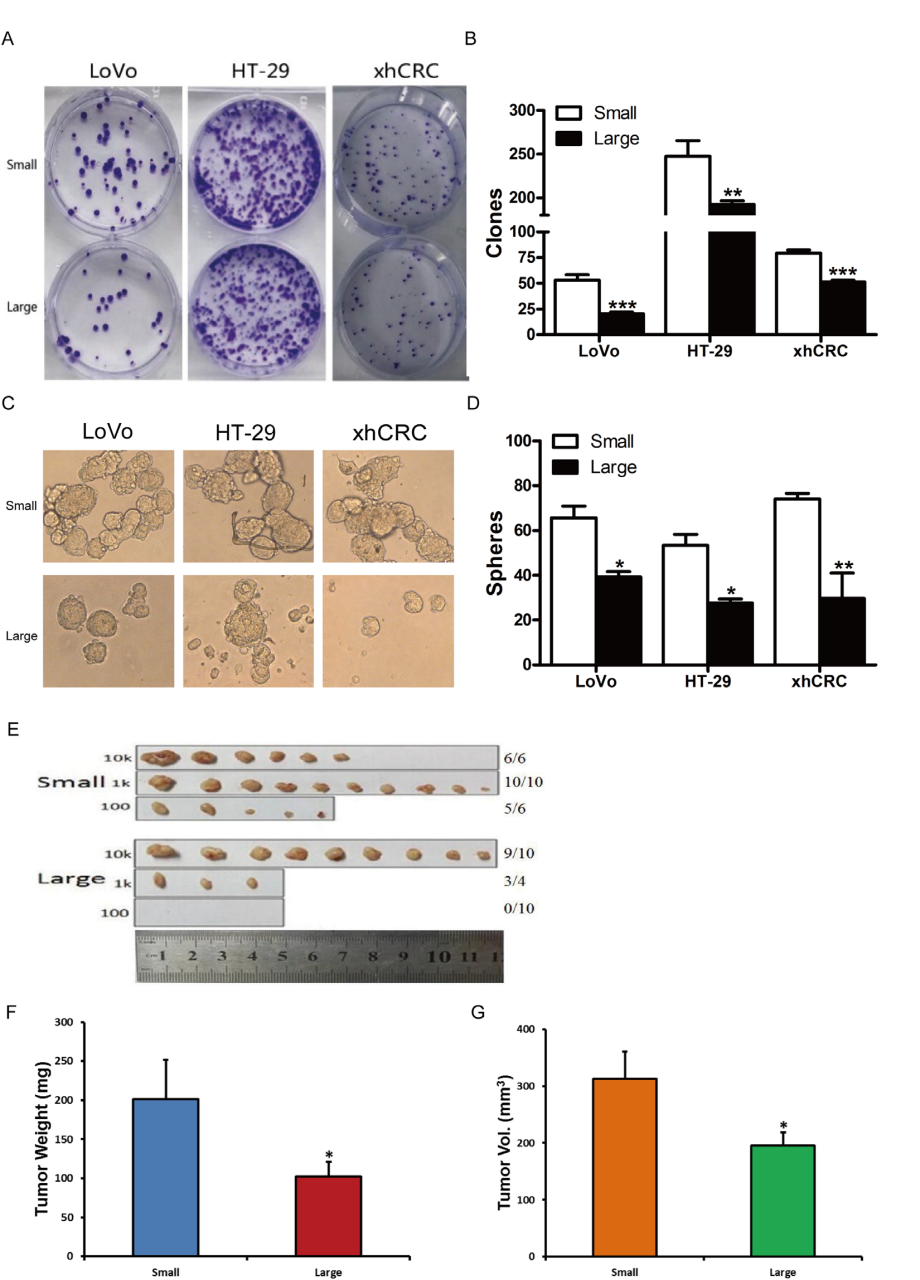
Small cells possess higher self-renewal than corresponding large cells in CRC **(A-B)** Clonal culture for sorted cells. Large- and small-sized subpopulations were sorted out in LoVo, HT-29 and xhCRC cells, and seeded in the plates. Holoclones were stained by 0.1% Crystal violet, and then photographed (A) and counted (B) 10 days later. Data are presented from three separate experiments. **(C-D)** Sphere formation assays for sorted cells. Large- and small-sized subpopulations were sorted out in LoVo, HT29 and xhCRC cells, and cultured in ultra-low attachment plates with stem cell medium. Spheres were photographed (C) and counted (D) 7days later. Data are presented from three separate experiments. **(E-G)** Small LoVo cells possess higher tumorigenicity. Sorted large and small LoVo cells were injected subcutaneously into BALB/c-nu female mice at 100, 1000, 10,000 cells per injection. 6 weeks after implanting, tumors were harvested. Tumor images, tumor incidence (E), tumor weights (F) and volumes (G) were shown. Data are presented as means ± SD, ^*^P< 0.05, ^**^P< 0.01, ^***^P< 0.001.

To test whether purified small CRC cells possess higher self-renewing capacity, we performed clonal culture. Purified small CRC cells (i.e., LoVo, HT-29 and xhCRC) formed more holoclones than the isogenic large cells (Figure 2A and 2B), suggesting that small CRC cells may harbor more tumor-initiating cells since holoclones were shown to enrich for CSCs [21]. In addition, we further conducted sphere formation assays for sorted large and small CRC cells. Purified small CRC cells formed more and larger spheres than the corresponding large cells (Figure 2C and 2D). Our results demonstrate that small cells exhibit higher self-renewing capacity *in vitro* than large CRC cells.

To investigate whether small CRC cells enrich for TICs, we conducted limiting dilution assays (LDAs). Expectedly, purified small LoVo cells demonstrated higher tumor-generating capacity (Table 1) (*P*<0.001). And furthermore, purified small LoVo cells developed larger tumors than large cells (Figure 2E–2G and Supplementary Figure 1). Together, our findings indicate that small CRC cells possess higher tumor-initiating capacity than large cells.

**Table 1:** Tumor-Initiating frequency of large and small CRC cells in Balb/c-nu mice

Cells	10k	Cell dose		Tumor-initiating frequency(95% interval)^a^	P value
		1k	100		
LoVo					
Small	6/6	10/10	5/6	1/141(1/48-413)	8.79e-12
Large	9/10	3/4	0/10	1/8470(1/3547-15593)	
HT-29					
Small	9/10	5/6	0/6	1/2468(1/1038-5871)	0.242
Large	9/10	2/10	0/6	1/4454(1/2238-8865)	

Note. Purified large and small CRC cells were implanted subcutaneously in female Balb/c-nu mice and all tumors were harvested 6 weeks post-implantation.

^a^ Tumor-initiating frequency was determined using Bioinformatics Division ELDA analyzer (http://bioinf.wehi.edu.au/software/elda/index.html).

### Small CRC cells are more quiescent and highly express CD133

**Figure 3:**
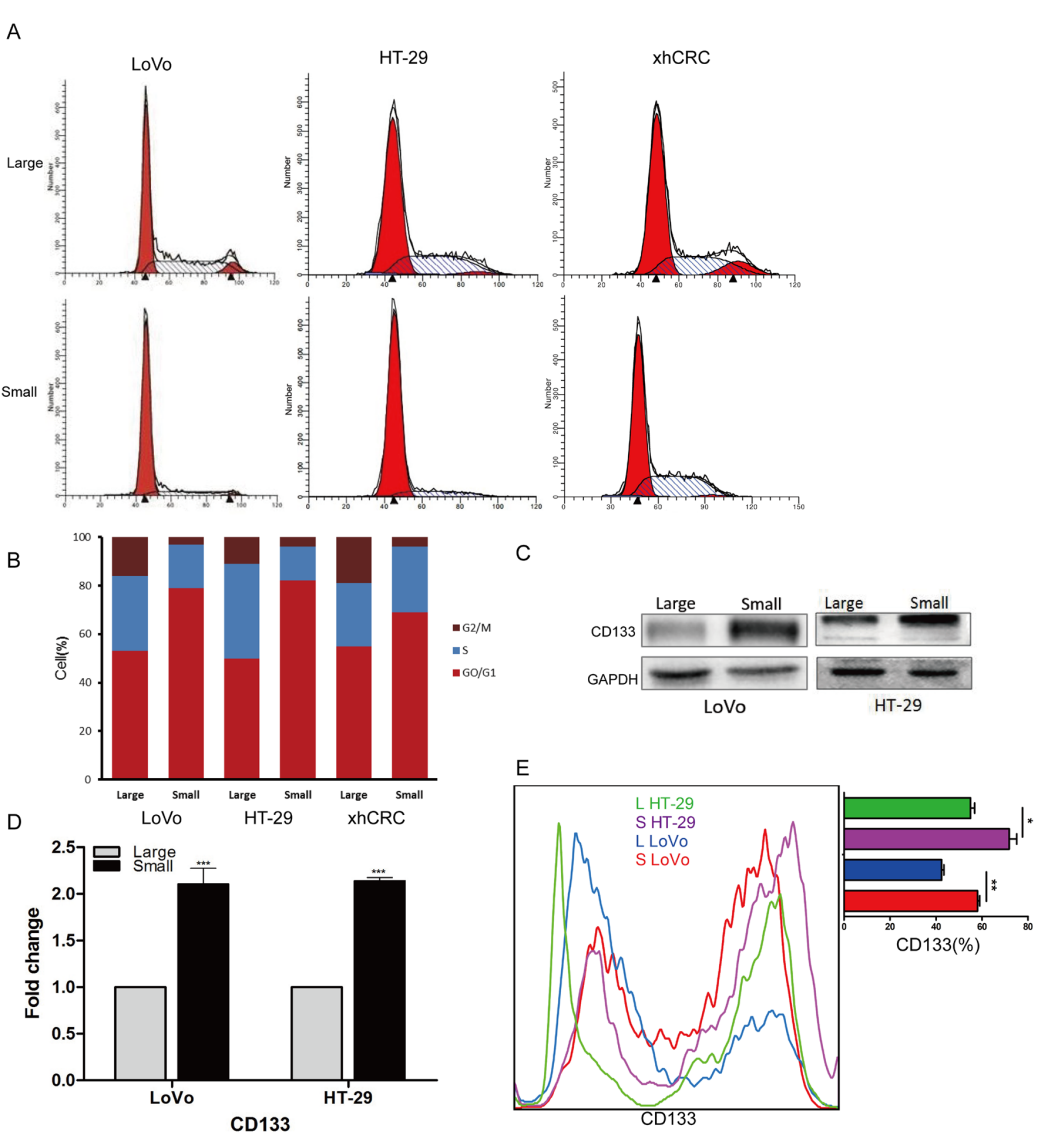
Small cells are slow-cycling cells, express CD133 **(A-B)** Cell cycle analysis of large and small cells. Small CRC cells (i.e., LoVo, HT29 and xhCRC) exhibit a higher percentage of cells in G0/G1 phase, while a lower percentage of S and G2/M phase than the large CRC cells. **(C-D)** Expression of CD133 in large and small cells was detected by western blotting and RT-qPCR. GAPDH was used as a loading control. **(E)** Expression of CD133 in large and small cells was analysis by FACS. Data are presented from triple experiments; mean ± SD, ^*^P< 0.05, ^**^P< 0.01, ^***^P< 0.001.

Studies have shown that TICs or CSCs may be more quiescent than committed differentiated cells [22–24]. To test whether sorted small-sized CRC cells are more quiescent, we performed cell-cycle analysis using FACS. We found that the percentage of small CRC cells in S and G_2_/M phases is smaller than the corresponding large CRC cells (Figure 3A and 3B), suggesting that small CRC cells enrich for quiescent cells. Recent studies have also shown that cell surface marker CD133 is used to prospectively enrich CSCs in CRC [20, 25, 26], therefore, we examined the expression of CD133 in purified small and large CRC cells using western blotting (Figure 3C), RT-qPCR (Figure 3D) and FACS (Figure 3E). Our results showed that, when compared to the large CRC cells (i.e., LoVo and HT-29 cells), small CRC cells expressed higher CD133 than the corresponding large CRC cells at the mRNA and protein level.

Collectively, these results suggest that purified small CRC cells are more quiescent and highly express CD133 at the mRNA and protein level.

### Small CRC cells possess high metastatic capacity

**Figure 4:**
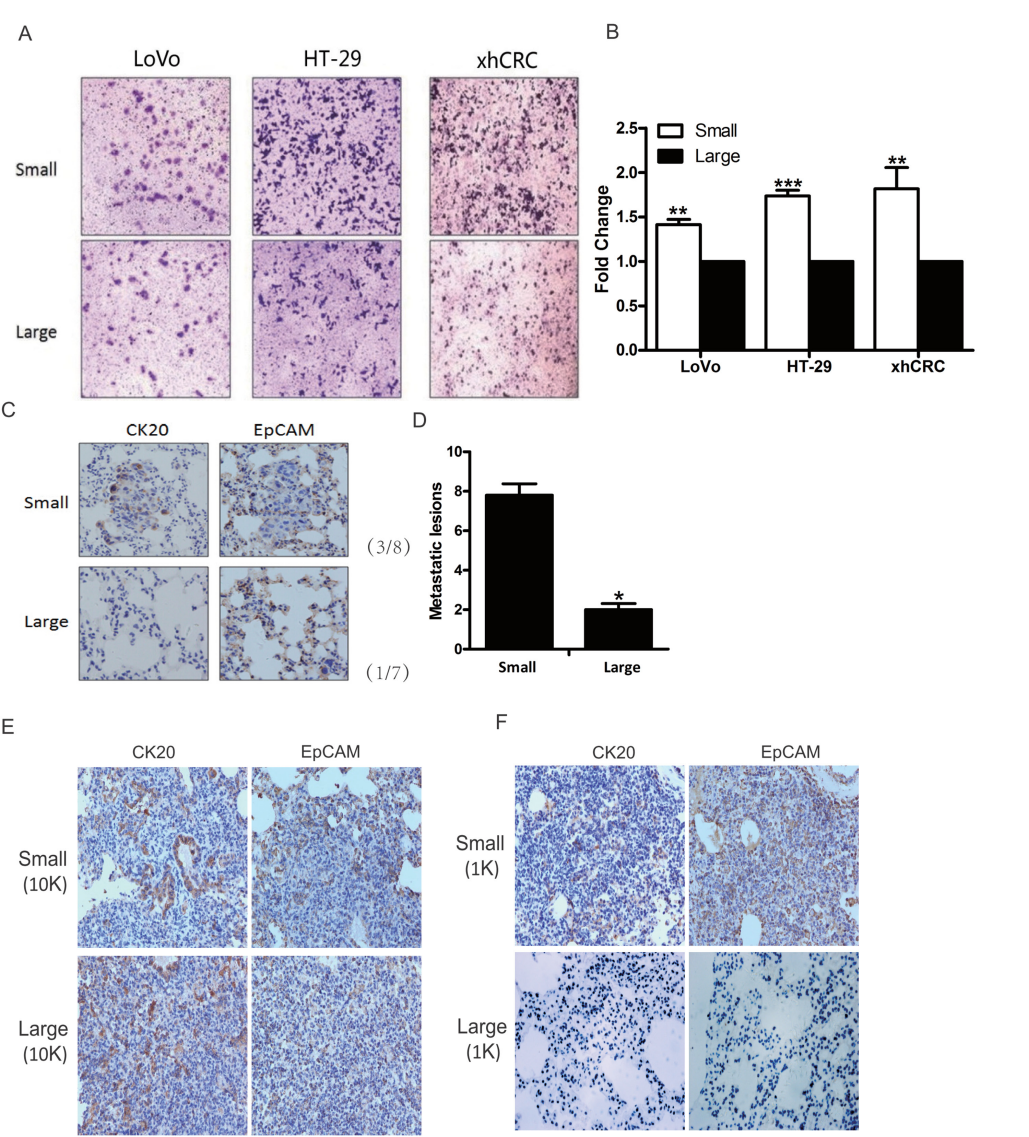
Small CRC cells possess higher metastatic capacity **(A-B)** Transwell assays. Large- and small-sized subpopulations were sorted out in LoVo, HT-29 and xhCRC cells, and seeded in transwell inserts covered with Matrigel. After 24 hours, cells migrated through Matrigel barrier were photographed (left panel) and calculated (right panel). Scale bars: 100 μm. Data are presented from triple experiments. **(C-D)** Sorted large and small LoVo cells were injected into the tail vein of NOD/SCID mice (n=6 per group). 8 weeks later, animals were scarified for examining metastatic lesions in the lungs. Expression of CK20 and EpCAM in metastatic lesions was examined by immunohistochemistry staining. Scale bars: 100 μm. **(E-F)** Purified large and small LoVo cells were implanted subcutaneously into BALB/c-nu female. 6 weeks later, the mice were scarified for harvesting the lungs to examine metastatic lesions. Implanted at 10K cell dose, both large and small LoVo cells formed metastatic lesions in the lungs of the mice, while at 1K cell dose, only small LoVo cells formed lung metastatic lesions. Expression of CK20 and EpCAM in metastatic lesions was confirmed by immunohistochemistry staining. Scale bars: 100 μm.(Mean ±SD, n =5, ^*^P< 0.05, ^**^P< 0.01, ^***^P< 0.001)

Recent studies have shown that TICs may also harbor metastatic cells [27–29], furthermore, small cells may be more easily to pass through the blood and lymphatic vessels due to their sizes. To evaluate whether small CRC cells possess higher metastatic capacity than the corresponding large cells, we first performed transwell invasion assay for sorted small and large CRC cells (i.e., LoVo, HT-29 and xhCRC). Interestingly, small CRC cells possessed higher invasion capacity than the isogenic large cells (Figure 4A and 4B).

Next, we examined metastatic potential using injection of purified small and large LoVo cells into tail veins of female NOD/SCID mice. Consistent with the findings in transwell invasion assay, small LoVo cells initiated more lung metastatic lesions than the corresponding large cells in NOD/SCID mice (Figure 4C and 4D), and furthermore, immunostaining confirmed that the metastatic cells were positive for CRC epithelial markers such as CK20 and EpCAM (Figure 4C). Interestingly, we found that only small LoVo cells-initiated subcutaneous tumors generated lung metastatic lesions, while large LoVo cells-initiated subcutaneous tumors failed to form metastatic lesions (Figure 4E and 4F).

Overall, these results clearly demonstrate that small CRC cells possess higher metastatic capacity than the corresponding large cells.

### YAP1 regulates self-renewing capacity and metastatic potential in small CRC cells

**Figure 5:**
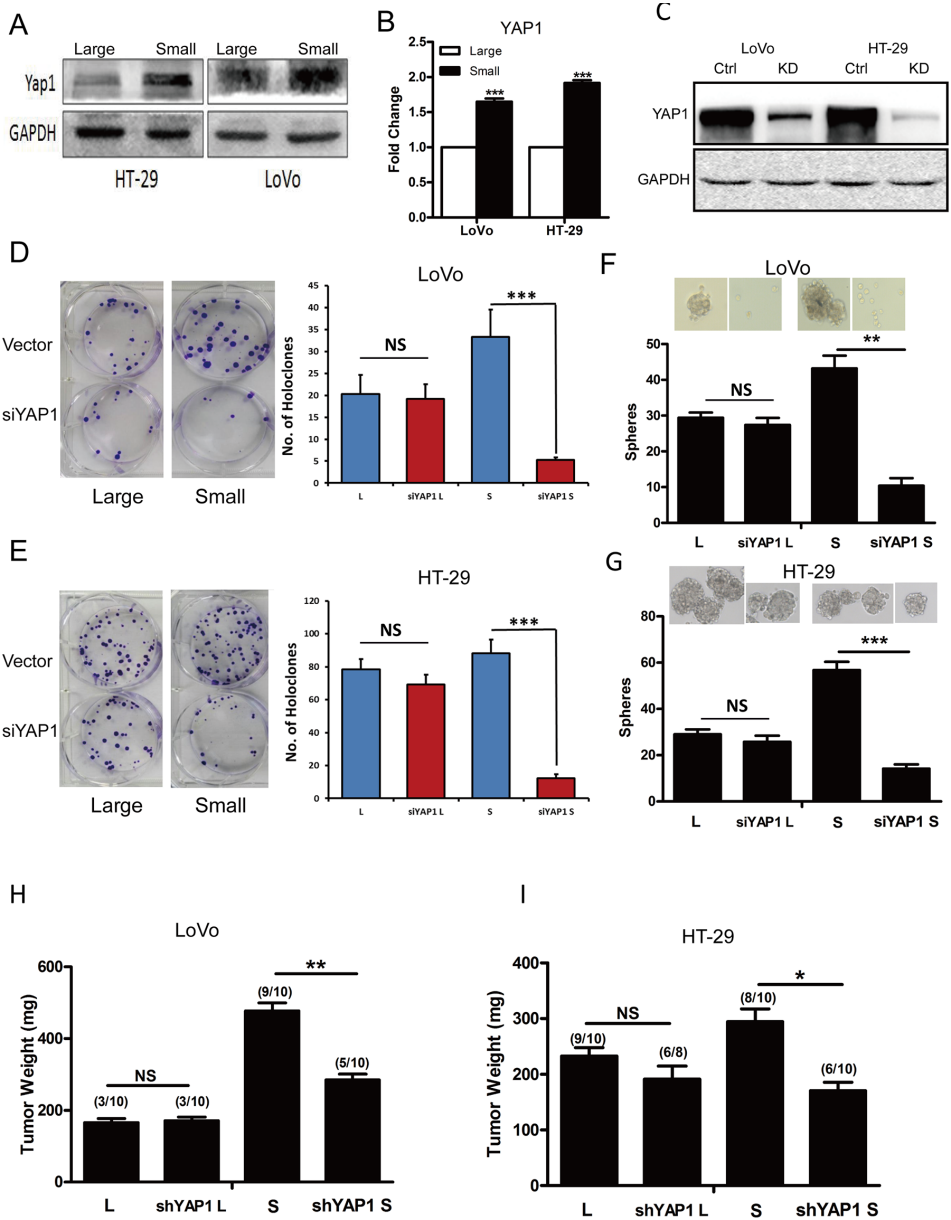
Down-regulation of YAP1 decreased holoclone-, sphere-forming capacity and invasive capacity in small CRC cells **(A-B)** Expression of YAP1 in large and small LoVo, HT-29 cells was detected by western blotting (A) and RT-qPCR (B). GAPDH was used as a loading control. Data are presented from triple experiments. **(C)** Knockdown of YAP1 in LoVo, HT-29 cells was measured by western blotting. GAPDH was used as a loading control. Data are presented from triple experiments. **(D-E)** Clonal culture for large and small LoVo (D), HT-29 (E) cells upon knocking down of YAP1. (L denotes large CRC cells, S denotes small CRC cells). Data are presented from triple experiments. **(F-G)** Sphere formation assay for large and small LoVo (F), HT-29 (G) cells upon knocking down of YAP1. Data are presented from triple experiments. **(H-I)** Tumor transplantation for large and small LoVo (H), HT-29 (I) cells upon knocking down of YAP1. Shown are tumor weights and incidence. Mean ± SD, ^*^P< 0.05, ^**^P< 0.01, ^***^P< 0.001.

Numerous studies have shown that multiple signaling pathways are involved in regulation of cell size, such as mTOR, Myc and Hippo signaling pathway [30–33]. However, Yap1 plays a significant role to mediate the cross-linking of Hippo and PI3K-TOR signaling pathways [34]. To explore whether YAP1 mediates the regulation of cell size in CRC cells, we first examined the expression of YAP1 in purified large and small CRC cells using western blotting and RT-qPCR analysis. Our findings revealed that purified small CRC cells (i.e., LoVo and HT-29 cells) expressed higher YAP1 than the large CRC cells at the protein (Figure 5A) and mRNA level (Figure 5B). We further applied RNAi-mediated approach to investigate whether YAP1 regulated the self-renewing capacity of small CRC cells. Knockdown of YAP1 in LoVo, HT29 cells was confirmed by western blotting (Figure 5C). Intriguingly, knockdown of YAP1 in purified small CRC cells (i.e., LoVo and HT-29 cells) inhibited holoclone-forming (Figure 5D and 5E) and sphere-forming capacity (Figure 5F and 5G) while there was no significant difference upon knocking down of YAP1 in purified large CRC cells. Moreover, parallel to YAP1 knock-down experiments, after treated with the YAP-TEAD inhibitor Verteporfin, holoclone-and sphere-forming capacity of small HT-29 and LoVo cells was significantly decreased, while there was no effect on that of corresponding large cells(Supplementary Figure 2). Importantly, consistent with *in vitro* results, purified small LoVo, HT29 cells displayed decreased tumor weight whereas there was no significant difference in purified large LoVo, HT29 cells upon knocking down of YAP1 (Figure 5H and 5I). These results indicate that YAP1 may increase the self-renewing capacity of small CRC cells whereas has no effects on that of large CRC cells.

**Figure 6:**
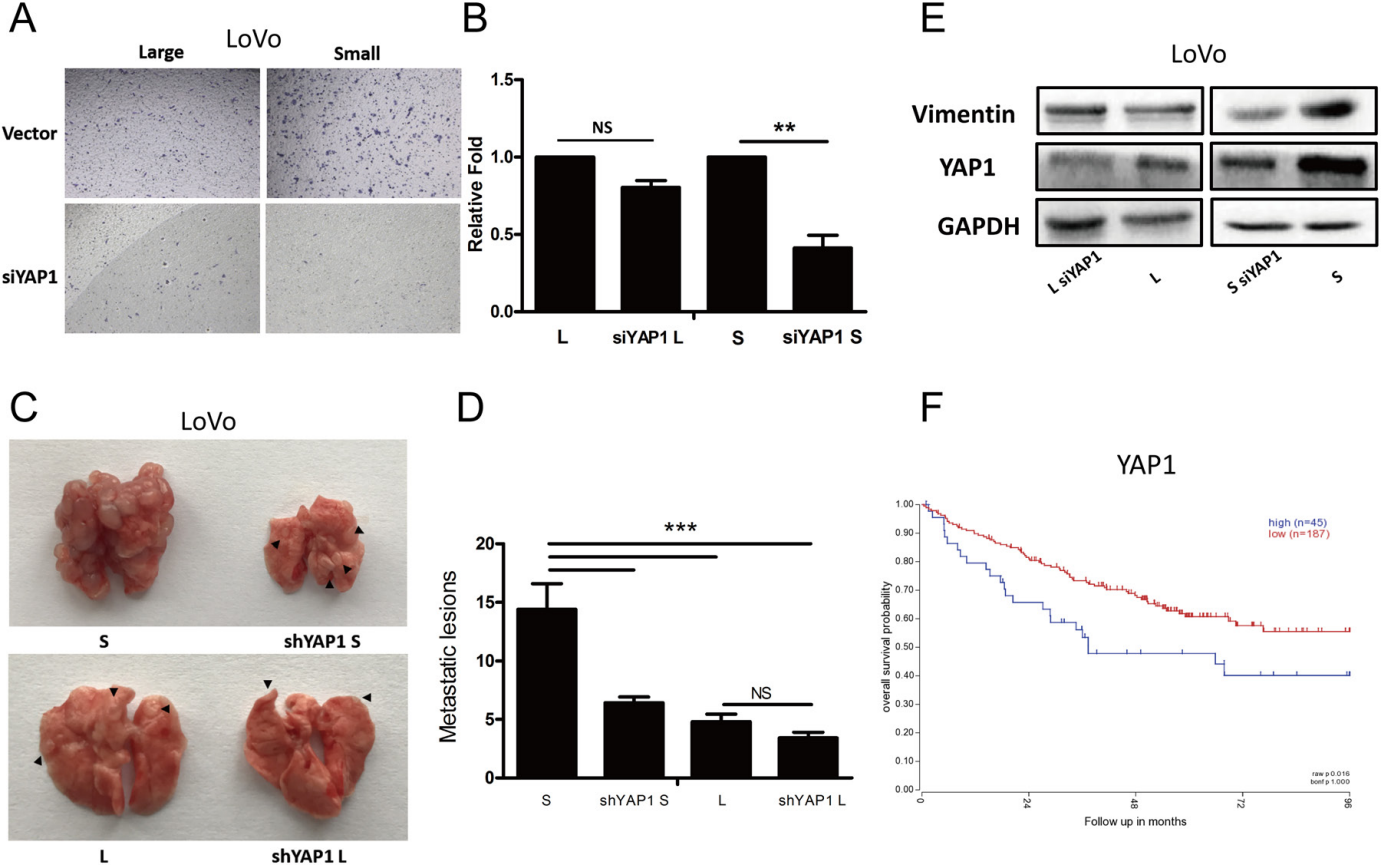
YAP1 regulates tumorigenicity and lung metastatic potential in small cells but not large CRC cells **(A-B)** Transwell assays for large and small LoVo cells upon knocking down of YAP1. (L denotes large CRC cells, S denotes small CRC cells) Scale bars: 100 μm. Data are presented as mean ± SD; ^**^P< 0.01. **(C-D)** Lung metastasis models of the effect of YAP1 knockdown. Representative images of lung metastases resulting from injection of large and small LoVo cells upon knocking down of YAP1 into NOD/SCID mice (n=6 per group). Data are presented as mean ± SD; ^***^P< 0.001. **(E)** Protein levels of E-cadherin, Vimentin, YAP1 and CD133 were analyzed using western blotting in large and small LoVo cells upon knocking down of YAP1. GAPDH was used as a loading control. **(F)** Kaplan-Meier analysis of the correlation of YAP1 with overall survival in colorectal cancer patients.

To investigate whether YAP1 mediate the metastatic potential, we first performed transwell invasion assay. Interestingly, knockdown of YAP1 significantly inhibited the migration capacity of small LoVo cells whereas had no effects on that of large LoVo cells (Figure 6A and 6B). Next, we further conducted the metastatic experiments for large and small LoVo cells. Consistent with the *in vitro* findings, small LoVo cells formed much less metastatic lesions upon knocking down of YAP1 whereas knockdown of YAP1 had no significant effects on large LoVo cells at metastatic potential (Figure 6C and 6D). In support of the point that epithelial-mesenchymal transition (EMT) is closely associated with metastasis of tumor cells [35], we found that in small LoVo cells not large cells, knockdown of YAP1 down-regulated the expression of vimentin, a key EMT protein (Figure 6E). We finally explored the correlation between the expression of YAP1 in tumors and clinical outcome in CRC patients. Using R2 database, we found that expression of YAP1 positively correlated with poor prognosis in CRCs (Figure 6F).

Together, our findings clearly reveal that down-regulated of YAP1 decreases self-renewing capacity and metastatic potential in small CRC cells and positively correlates with poor prognosis in CRC.

## DISCUSSION

In the present study, our findings show that, at least in two widely used CRC cell lines (i.e., LoVo and HT-29 cells) and one PDX (i.e., xhCRC), small-sized CRC cells enrich for TICs or CSCs, possess higher metastatic capacity than the corresponding large-sized cells. Importantly, our results show that YAP1 is responsible to regulate the self-renewing capacity and metastatic potential of small CRC cells while it has little effects on the corresponding large CRC cells. And interestingly, our findings also show that YAP1 positively correlates with the poor prognosis in CRCs.

Currently, most of the studies use flow cytometry to sort out the different-sized cells [36–38]. This tactic has both pros and cons [38, 39]. The sorting gate may involve false positive and negative results, as a minor portion of small cells may be cell debris, and large cells may contain few adherent cells, in order to avoid these problems, we chose optimal gate more intuitively, used cell strainer to remove adherent cells, stained cells with propidium iodide to distinguish dead cells. Using forward scatter could be the indirect way to measure cell size and sort out large and small cells conveniently without fluorescent stain, but different flow cytometry machine may provide different detecting results. Consequently, we increased the purity by multiple sorting and confirmed the given sorted cells under microscope (Figure 1C–1E).

CRCs contain different-sized cancer cells, the cells located in different cell cycle phase possess different cell sizes during cell cycle progression [10]. In same cell cycles, the cells in G_0_/G_1_ phase are generally smaller than those in S phase, and are much smaller than the ones in G_2_/M phase. And moreover, in different cell cycles, the cells even in same cell cycle phases have varied cell sizes. Consistent with this point, in the current study, our results clearly demonstrated that some of small CRC cells located in G_0_/G_1_-phase cells, and some of them were G_2_/M-phase cells although the percentage of G_0_/G_1_-phase cells was larger than that in large CRC cells, implying that there are other factors regulating the cell size besides cell cycle.

The relationship between cell size and stemness has been of great interest to researchers. Generally, stem cells are more quiescent, possessing the capacity for self-renewal and the multilineage potential [40], and cell division is associated with cell size and age [41]. In our study, we find that the small CRC cells can form more holoclones and spheres, as well as display higher stem cell frequencies in limiting dilution assays (Figure 2), suggesting that small cells harbor more TICs. This is in agreement with another study that small cells in the human epidermoid carcinoma cell line A431, which derived from non-adherent spheres, exhibited a stronger ability to form spheres and higher tumorigenicity in mice [42]. In addition, we found that more small CRC cells were in the G_0_/G_1_ phase (Figure 3A and 3B), a relatively quiescent state. Similarly, in human corneal epithelial cells, small-sized cells retained more BrdU in label-retaining assay, implying that the cells are more quiescent [43]. Moreover, we and others have identified that the stem cell markers CD133 can be used to enrich CSCs [20], here, at the mRNA and protein level, we observed that small CRC cells preferentially express CD133 (Figure 3C–3E).

Recent discoveries have provided supports that TICs take part in metastasis [29]. Nuclear rupture [44] and cell deformation [45] are also found to be involved in metastatic process. Compared to large-sized cells, small-sized cells might be more easily to penetrate through confining spaces in metastatic process since large cells may easily be caused to more nuclear rupture and hardly be deformation. Consistent with the studies, our data indicate that small CRC cells exhibit higher invasion capacity *in vitro* and metastatic capacity *in vivo* compared with large cells (Figure 4).

YAP1 regulates cell size and tissue growth [46]. It has previously been shown that YAP1 affects the stemness of tumor cells and promotes tumor metastasis [47]. Studies have demonstrated that Sox2 can enhance the function of YAP1 thus maintain the stemness of tumor cells [48]. Moreover, in ovarian cancer, YAP1 may promote self-renewal of TICs [49]. In addition, studies have shown that VEGF-C increases YAP1, down-regulates the expression of slug, and thus enhances the self-renewing capacity and metastatic potential of tumor cells [50]. Consistent with these observations, we found that the expression of YAP1 in small colorectal cancer cells was higher than the corresponding large cells, and furthermore, knockdown of YAP1 decreased the stemness (Figure 5D–5I) and metastatic capacity (Figure 6A–6D) of small CRC cells, while those were little affected in large CRC cells upon knocking down of YAP1.

In summary, our results provide rationale that novel therapeutics targeting YAP1 in small CRC cells should be developed to gain maximal clinical benefits.

## MATERIALS AND METHODS

### Cell lines and culture

Human colon cancer cells (HCT116, SW48, LoVo and HT-29) were purchased from American Type Culture Collection (ATCC), xhCRC were established and passaged as previously described [18]. In general, primary colorectal tumors were mechanically dissociated and digested in DMEM medium contained collagenase IV (Invitrogen, California, USA), hyaluronidase (Sigma, St. Louis, USA). Single tumor cells were obtained after filtered through a cell strainer (BD Falcon, CA, USA), incubated in red blood cell lysis buffer (eBioscience, California, USA), using to eliminate red blood cells. To establish xenograft tumor model, cells were implanted into female NOD/SCID mice subcutaneously. All the cell lines were grown in high glucose DMEM (Invitrogen, California, USA) with 10% fetal bovine serum (Gibco, NY, USA), 100μg/ml penicillin, and 100μg/ml streptomycin in a 5% CO2 incubator at 37°C.

### Flow cytometry

All cells were first passed through a 30μm cell strainer to remove adherent cells, then large and small cells were sorted by FACS (Aria II BD Biosciences, CA, USA). Generally, based on forward scatter (FSC), top 15% and bottom 15% gated cells were sorted out. Debris were removed by gates in the light scatter (LSC) versus FSC diagrams. To distinguish dead cells, cells were stained with propidium iodide (Sigma, USA). 3 cycles of sorting were performed in order to maximize purify large and small CRC cells. For cell cycle analyses, cells were fixed in 70% cold ethanol at 4°C for 12 hours, then incubated with RNAse A working solution (0.25 mg/ml) and PI working solution(50μg/ml) for 30 min before FACS was performed.

### Clonal culture and sphere formation assay

Clonal culture and sphere formation assay were performed essentially as described previously [19]. To analyze clonal formation ability, 200 or 300 purified large and small cells were seeded in a six-well plate. Clones were stained and counted after 10 days of growth. For sphere formation assays, purified large and small cells were plated 100 or 200 cells per well in 24-well ultra-low attachment plates, after cultured in serum-free medium for one week, spheres> 50μm were counted. For Verteporfin (VP) (Sigma, St. Louis, USA) treatment, cells were treated in the dark with a concentration of 5μM. Cellular toxicity of VP (5μM) was < 8% in LoVo cells and 5% in HT-29 cells, respectively.

### Animal studies

Female NOD/SCID mice and BALB/c-nu mice (4–6 weeks of age) were purchased from Beijing HFK Bioscience CO., LTD. (Beijing, China). All experiments were strictly performed according to the relevant national and international guidelines, and approved by the Huazhong University of Science and Technology Animal Care Committee. For xenograft studies, cells suspended in PBS were mixed with the same volume Matrigel (BD Biosciences, CA, USA), then injected subcutaneously into BALB/c-nu mice at the volume of 100 μl [20]. 6 weeks later, tumors were harvested, tumor volumes and weight were examined. For metastasis assay, 5 x10^5^ cells suspended in 100μl PBS were injected into the tail vein of NOD/SCID mice. Animals were scarified 8 weeks later, metastatic burden was recorded.

### Transwell invasion assays

Cells were resuspended in 200μ DMEM medium without fetal bovine serum, then incubated into transwell chamber (8 μm pores; Corning, NY, USA) covered with Matrigel and 650μl DMEM medium (15% FBS) was added to the bottom of the chamber. 24hours later, cells on the lower surface of transwell insert were stained with 0.1% crystal violet and photographed by microscope (Olympus). A total of 10 randomly selected fields in each transwell insert were evaluated.

### Immunohistochemistry

Basic procedures for immunohistochemistry of formalin-fixed paraffin-embedded sections were performed as previously described [19]. Eight fields were chosen in one slide by two experienced pathologists. Antibodies used for immunohistochemistry are as follows: anti-CK20 (Cell Signaling Technology, 13063, 1:100), anti-EpCAM (MiltenyiBiotec, 130-098-793, 1:100). Antibodies used for western blot are as follow: anti-GAPDH (abcam, ab9484, 1:1,000), anti-CD133 (MiltenyiBiotec, 130-092-395, 1:250), anti- E-Cadherin (CST #3195, 1:1,000), anti- Vimentin (CST #5741, 1:1,000), anti-YAP1 (abcam, 1674Y, 1:1,000).

### qRT - PCR

Basic protocols for RT-PCR analyses have been described [18]. Quantitative RT-PCR was performed using the SYBR-Green PCR master mix (Thermo Scientific, K0221) on ABI PRISM 7300 Sequence Detection System (Applied Biosystem). The PCR amplification is performed at the following conditions: 95°C for 10 min, followed by 40 cycles at 95°C for 15 s and 60°C for 1 min. Primers for the gene expression analysis are as follows: GAPDH: 5’-TCGTGGAAGGACTCATGACC-3’ (forward) and 5’-TCCACCACCCTGTTGCTGTA-3 (reverse);CD133:5’- TTCTTGACCGACTGAGA CCCA-3’ (forward) and 5’- TCATGTTCTCCAACGCC TCTT-3’ (reverse);YAP1:5’- TAGCCCTGCGTAGCCA GTTA-3’ (forward) and 5’- TCATGCTTAGTCCACTGT CTGT-3’ (reverse)

### Cell transfection

Cells were transiently transfected with 20μM scrambled siRNA or YAP1 siRNA designed and purchased from Ribobio (Guangzhou, China). Lipofectamine 2000 reagent (Invitrogen) was used for transfection according to the instructions provided by the manufacturer.

YAP1-shRNA lentivirus were purchased from Shanghai SBO Medical Biotechnology (Shanghai, China). Cells were seeded in 6-well plates 5 x10^4^ per well 24h before transfection and infected with YAP1-shRNA lentivirus or vector for 3 days at MOI of 25.

### Bioinformatics and statistics analysis

Data were expressed as mean ± SD. Statistical differences was compared using Student's t-test and one-way ANOVA analysis. Survival analysis was performed using the Kaplan-Meier method by R2 web platform (http://r2.amc.nl). ^*^p<0.05, ^**^p<0.01 and ^***^p<0.001; NS represents no significant differences.

## SUPPLEMENTARY MATERIALS FIGURES



## Abbreviations

CRC: colorectal cancer
TICs: tumor-initiating cells
FACS: fluorescence-activated cell sorting
FSC: forward scatter
YAP1: yes-associated protein 1
xhCRC: human patient-derived CRC xenograft cell line
LDAs: limiting dilution assays
EMT: epithelial-mesenchymal transition
