# Supplementary material for: Small-sized colorectal cancer cells harbor metastatic tumor-initiating cells
Source: Oncotarget. 2017 Nov 11;8(64):107907–19. doi: 10.18632/oncotarget.22392 (PMC5746114; doi:10.18632/oncotarget.22392)
Supplement: Supplementary file 1 [file oncotarget-08-107907-s001.pdf]

Small-sized colorectal cancer cells harbor metastatic tumor-initiating cells

SUPPLEMENTARY MATERIALS

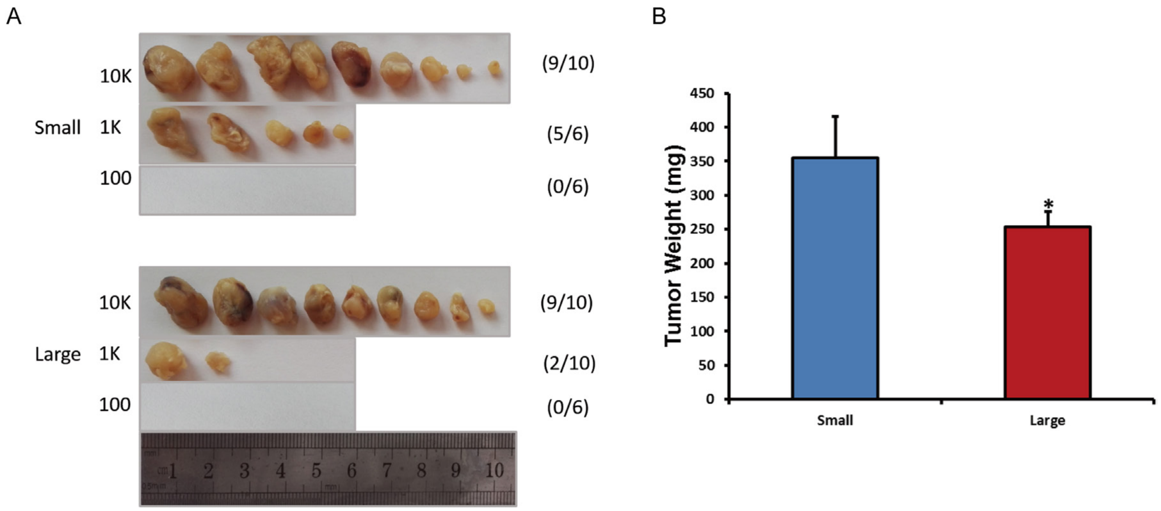

Supplementary Figure 1: Small HT-29 cells possess higher tumorigenicity. **(A-B)** Sorted large and small HT29 cells were injected subcutaneously into BALB/c-nu female mice at 100, 1000, 10,000 cells per injection. 6 weeks after implanting, tumors were harvested. Tumor images, tumor incidence (A), tumor weights (B) were shown.

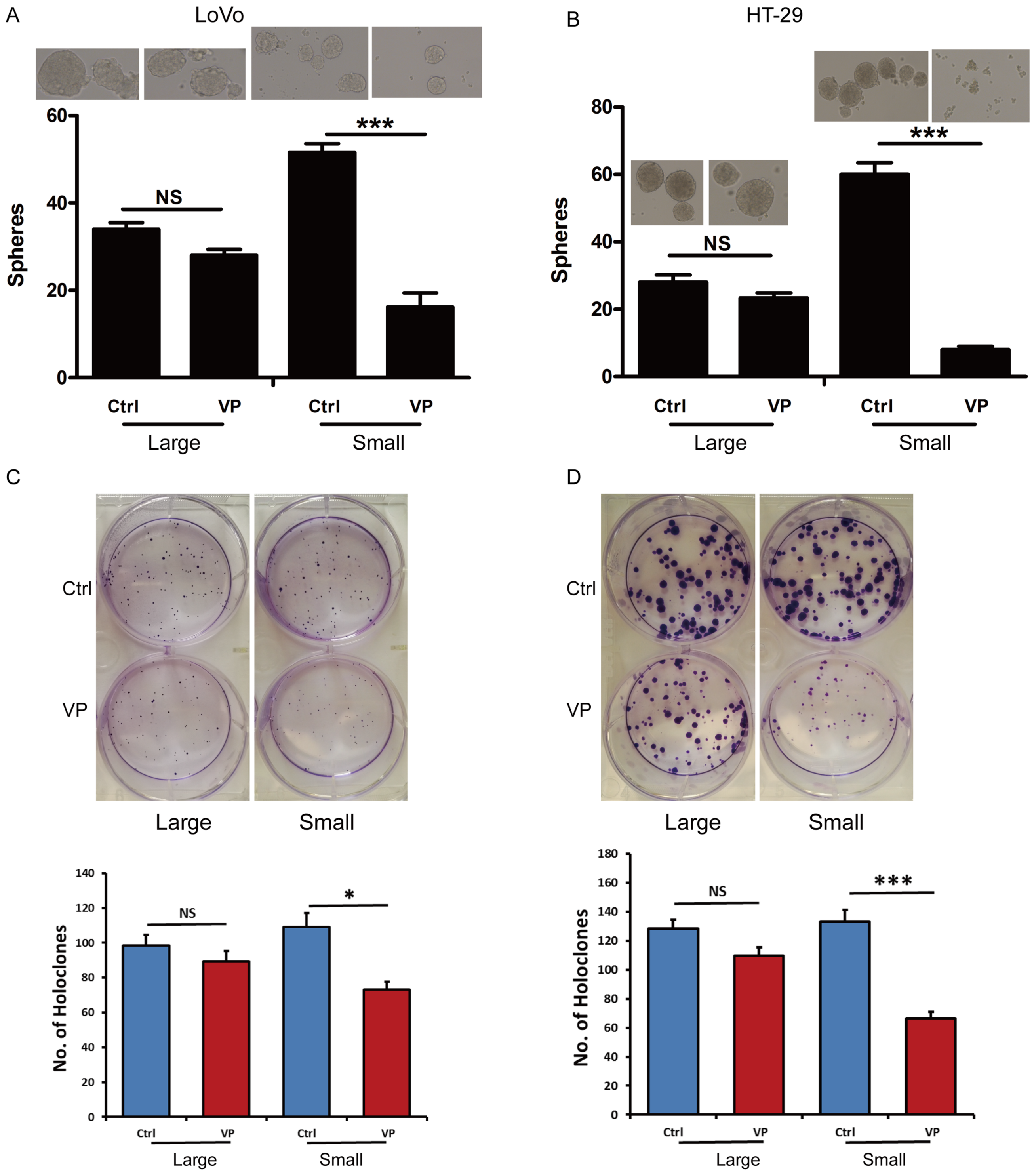

Supplementary Figure 2: Verteporfin inhibits small LoVo and HT-29 cell holoclone- and sphere-forming capacity. **(A-B)** Clonal culture for large and small LoVo (A), HT-29 (B) cells treated with Verteporfin. (L denotes large CRC cells, S denotes small CRC cells). Data are presented from triple experiments. **(C-D)** Sphere formation assays for large and small LoVo (C), HT-29 (D) cells treated with Verteporfin. Data are presented from triple experiments.
